# Influence of sugar concentration on the vesicle compactness, deformation and membrane poration induced by anionic nanoparticles

**DOI:** 10.1371/journal.pone.0275478

**Published:** 2022-09-29

**Authors:** Sharif Hasan, Mohammad Abu Sayem Karal, Salma Akter, Marzuk Ahmed, Md. Kabir Ahamed, Shareef Ahammed

**Affiliations:** 1 Department of Physics, Bangladesh University of Engineering and Technology, Dhaka, Bangladesh; 2 Radiation, Transport and Waste Safety Division, Bangladesh Atomic Energy Regulatory Authority, Dhaka, Bangladesh; Université Sorbonne Paris Nord: Universite Sorbonne Paris Nord, FRANCE

## Abstract

Sugar plays a vital role in the structural and functional characteristics of cells. Hence, the interaction of NPs with cell membranes in the presence of sugar concentrations is important for medicinal and pharmacological innovations. This study integrated three tools: giant unilamellar vesicles (GUVs), anionic magnetite nanoparticles (NPs), and sugar concentrations, to understand a simplified mechanism for interactions between the vesicle membranes and NPs under various sugar concentrations. We focused on changing the sugar concentration in aqueous solution; more precisely, sucrose inside the GUVs and glucose outside with equal osmolarity. 1,2-dioleoyl-*sn*-glycero-3-phospho-(1′-*rac*-glycerol) (sodium salt) (DOPG) and 1,2-dioleoyl-*sn*-glycero-3-phosphocholine (DOPC) were used to prepare the charged membranes of 40mole%DOPG/60mole%DOPC-GUVs, whereas only DOPC was used to prepare the neutral membranes. Phase contrast fluorescence microscopy shows that the adherence of 18 nm magnetite NPs with anionic charge depends on the sugar concentration. The alterations of GUVs induced by the NPs are characterized in terms of i) vesicle compactness, ii) deformation, and iii) membrane poration. The presence of sugar provides additional structural stability to the GUVs and reduces the effects of the NPs with respect to these parameters; more precisely, the higher the sugar concentration, the smaller the alteration induced by the NPs. The differences in NPs effects are explained by the change in the type of interaction between sugar molecules and lipid membranes, namely enthalpy and entropy-driven interaction, respectively. In addition, such alterations are influenced by the surface charge density of the lipid bilayer. The surface pressure of membranes due to the adsorption of NPs is responsible for inducing the poration in membranes. The differences in deformation and poration in charged and neutral GUVs under various sugar concentrations are discussed based on the structure of the head of lipid molecules.

## 1. Introduction

Nanoparticles (NPs) of sizes less than 100 nm have been used in several biomedical applications such as cellular imaging [1], gene delivery [2], cancer treatment [3, 4], and targeted drug delivery [5–7]. In contrast, such NPs show antibacterial and anticarcinogenic properties [8–13] along with cytotoxic effects [14]. The adverse effect of NPs on human health occurs due to the entry of NPs into the body from environmental pollutants [15–19], medical implants, MRI contrast agents, insecticides, and food product processing particles [20–22], resulting in the discomfort of the cardiovascular and respiratory systems [23, 24]. One of the main reasons for this discomfort is the interaction of NPs with biomembranes and the changes in their biophysical and biochemical properties [25–27]. Magnetite NPs can be found from burning fuel in the iron industry, printer toners, stoves, etc. Plenty of magnetite NPs were identified in the human brain, which are prolific in urban areas. The presence of magnetite NPs in the brain causes neurodegenerative diseases such as Alzheimer’s [28, 29]. As previously stated, NPs interact with cell membranes, and thus the stability of the membranes in aqueous solution is influenced by various ingredients such as salt concentration and lipid bilayer surface charge density [30]. Sugar, in addition to these elements, plays an important role in the structural and functional properties of biomembranes under various conditions [31–33]. In addition to its critical role in cellular regulation, it has a wide range of applications in medical and industrial research [34, 35]. Sugar influences the spontaneous curvature of vesicles [36], specific capacitance of membranes [37], and bending rigidity of membranes [38–40]. Recently, we investigated the effects of sugar concentration on electroporation in lipid vesicles for the possible application of electroporation technology in cancer/tumor ablation [41]. Therefore, a better understanding of how NPs interact with cells in the presence of various sugar concentrations is vital for medicinal and pharmacological inventions. Cell-sized giant unilamellar vesicles (GUVs) have been used to investigate the interaction of membrane active agents (e.g., peptides, toxins) with lipid membranes [42–45]. GUVs have also been used to study the formation of pores using mechanical tension and electric field [46, 47].

The bending rigidity of lipid membranes changes with sugar concentration [38, 40]. Sugar molecules strongly interacted with the lipid membranes and inserted between the head group of phospholipids at relatively lower sugar concentrations [48]. This insertion lowered the lipid head group area by increasing the surface pressure. In the aqueous phase, a portion of the carbohydrate molecules accumulates in the membrane, forming a kind of additive [36, 49].

At lower sugar concentrations, an enthalpy-driven attractive interaction between sugars and the lipid head group was considered, while at higher sugar concentrations, an entropy-driven repulsive interaction was considered [50, 51].

There is a debate about transporting NPs into mammalian cells. NPs have entered using endocytosis pathway inhibitors [52], but the situation is not entirely obvious for erythrocytes [53]. The interaction of NPs with membranes involves hydrophobic mismatch effects [54], chain stretching of the lipids near the site of interaction [55], spontaneous curvature of membranes [56], changes in lipid packing [57], and pearling [58]. Various aspects of the interaction of NPs with lipid membranes were considered [59]. Magnetic NPs with a cationic core-shell tended to bind to positively charged bilayers [60]. Shape change (protrusion, pearling) of neutral GUVs was observed in the presence of encapsulating cationic NPs [58]. Membrane deformation occurs due to the binding of NPs with membranes [60, 61]. Recently, the interaction of anionic magnetic NPs (same as used here) with charged and neutral membranes in the presence of a fixed sugar concentration has been investigated [30], in which the interaction of NPs is explained based on the key role of the dipoles of DOPC lipids. As previously stated, sugar plays an important role in the structural and functional aspects of the lipid bilayer; thus, the question arises how sugar influences the NPs-induced vesicle compactness, vesicle deformation, and lipid membrane poration. These are the main goals of this investigation. As phosphocholine (PC) lipids are available in human cells, both charged and neutral vesicles prepared by PC membranes have been considered for quantification of compactness, fraction of deformed GUVs, and fraction of pore formed GUVs.

## 2. Materials and methods

### 2.1 Chemicals and reagents

Negatively charged lipid 1,2-dioleoyl-*sn*-glycero-3-phospho-(1′-*rac*-glycerol) (sodium salt) (DOPG) and neutral lipid 1,2-dioleoyl-*sn*-glycero-3-phosphocholine (DOPC) were purchased from Avanti Polar Lipids Inc. (Alabaster, AL). Bovine serum albumin (BSA), Piperazine-1, 4-bis (2-ethanesulfonic acid) (PIPES), O,O′-Bis (2-aminoethyl) ethyleneglycol-*N*,*N*,*N*′,*N*′,-tetraacetic acid (EGTA) were purchased from Sigma-Aldrich (Germany). Ferric chloride anhydrous (FeCl_3_), ferrous chloride tetra hydrate (FeCl_2_·4H_2_O), sucrose, and glucose were purchased from Merck, Germany.

### 2.2 Ipomoea aquatica leaf extracts mediated NPs

The green synthesis method was used to synthesize the NPs with a size of 18 nm as reported in our previous paper [62]. The synthesized particles had a cubic inverse spinel type phase. The zeta potential of the NPs was obtained at −21.3 mV [30]. *Ipomoea aquatica* (widely known as water spinach) was bought from Palashi market, Dhaka, Bangladesh. It is also available in any market in Bangladesh. At first, 60 g *Ipomoea aquatica* leaves paste was mixed into 400 mL deionized water and heated at 80°C for 4 hours at 800 rpm. 20 mL of 0.05 M FeCl_2_·4H_2_O and 20 mL of 0.10 M FeCl_3_ were mixed together at 60°C using 800 rpm and after 10 min, 5 mL leaf extract was added into the mixture, and after another 10 min 100 mL of 0.10 M NaOH solution was added to the mixture. During addition of NaOH, the nanoparticles were formed in the colloidal solution. The biomolecules of leaf extracts acted as stabilizers as well as reducing agents to prepare NPs using FeCl_3_ and FeCl_2_⋅4H_2_O as precursors.

The NPs were accumulated at the bottom of the glass beaker using a bar magnet, and the upper solution of NPs was discarded. The collected NPs were kept in a drier at 60°C for 2–3 days for fine drying. We prepared fine powders from dried NPs using a hand mortar. A higher concentration (0.20 mg/mL) of NPs was prepared in buffer (for charged membranes) or in MilliQ (for neutral membranes), which was then diluted to prepare 0.006, 0.01, and 0.013 mg/mL NPs solutions. The 100 μL NPs were then mixed with 200 μL suspension of purified GUVs in a microchamber (total 300 μL) for interacting the NPs with membranes. Hence, the corresponding effective NPs concentrations in the chamber were 2.00, 3.33, and 4.67 μg/mL.

### 2.3 Synthesis of lipid membranes of GUVs

Among the various methods to prepare the GUVs [63], we followed the well-known natural swelling method [64]. Charged membranes of 40%DOPG/60%DOPC-GUVs (where % indicates mole %) and neutral membranes of 100%DOPC-GUVs (i.e., DOPC-GUVs) with 1 mM concentration of DOPG and DOPC lipids (total volume 200 μL) were taken into a 4.5 mL glass vial individually. The samples were dried with a gentle flow of nitrogen gas to produce a thin, homogeneous lipid film. The vials were then kept in a vacuum desiccator for 12 hours. 20 μL MilliQ water was added into the vial for pre-hydration at 45°C for 8 min. The 40%DOPG/60%DOPC-GUVs and DOPC-GUVs were incubated for 3.0 hours at 37°C with 1 mL PIPES buffer (10 mM PIPES, 150 mM NaCl, pH 7.0, 1mM EGTA) and MilliQ water containing various concentrations of sucrose, respectively. Using these procedures, a population of charged and neutral GUVs were formed, which contained various concentrations of sucrose solution inside. To prepare the fluorescent probe (calcein) encapsulated GUVs, we used 1 mM calcein with various concentrations of sucrose in buffer (for charged vesicles) and MilliQ (for neutral vesicles) for incubation of the suspension of vesicles. Membrane filtering method was used to get the purified suspension of GUVs [65, 66] with outside solution of 40%DOPG/60%DOPC-GUVs and DOPC-GUVs were PIPES buffer and MilliQ containing various concentrations of glucose, respectively. Four different concentrations of sugar, i.e., *c* = 50, 100, 200 and 300 mM were used in these investigations. The concentration values refer to the concentrations of both sucrose (inside the GUV) and glucose (outside the GUV), which were kept equal to avoid any osmotic effect. After purification, 200 μL purified GUVs suspension was taken into a microchamber of volume 300 μL and then 100 μL NPs was added into the suspension. The effective NPs concentrations in the microchamber were 2.00, 3.33, and 4.67 μg/mL. The microchamber was prepared by inserting a U-shaped silicone rubber spacer onto a glass slide. We did not measure the concentration of GUVs but measured NPs concentrations. To remove the strong attraction between the glass surface and the membranes, the microchamber and the glass surface were coated with 0.10% (w/v) BSA solution. An inverted phase contrast fluorescent microscope (Olympus IX-73, Japan) with a 20× objective at 25 ± 1°C was used to observe the GUVs. The images were recorded using a charge-coupled device (CCD) camera (Olympus DP22, Japan) with a recording speed of 25 frames per second. The size of GUVs was measured using the cellSens Dimension (Ver. 3.2) PC software (Olympus Corporation, Japan).

### 2.4 NPs-induced compactness of GUVs

To investigate the NPs-induced compactness (*C*_*om*_) of GUVs, 100 μL NPs was added to 200 μL purified GUVs. We focused a GUV during the NPs interaction. The dynamics of that GUV was recorded using a charge-coupled device camera connected with phase contrast mode in the microscope. The time-dependent compactness and sugar concentration-dependent compactness for charged and neutral GUVs were determined. Compactness quantifies the change of the shape of GUVs [67] that can be directly measured using appropriate software. MATLAB image processing toolbox was used to calculate the *C*_*om*_ for each deformed image. The compactness is defined as follows [30]:

$$C_{om}=\frac{P^{2}}{4\pi S_{\mathrm{cr}}}$$ (1)

where *P* is the perimeter and *S*_cr_ is the image cross section area of a GUV. The minimal value of *C*_*om*_ is equal to 1.0 for a perfect circle while its value increases with any deviation. The degree of deformation of a GUV is determined by the value of compactness.

### 2.5 NPs-induced fraction of deformed GUVs and fraction of pore formed GUVs

The fraction of deformed GUVs (*Fr*_d_) and the fraction of pore formed GUVs (*Fr*_p_) were calculated by measuring the probability of deformation and poration among all the measured GUVs. Here, we explain how to calculate these fractions. 100 μL NPs were interacted with 200 μL purified GUVs suspension. During interaction of NPs with GUVs, the images of vesicles were taken at time 0, 10, 20, 30, 40, 50 and 60 min by keeping the focus at a fixed position. Then, similar experiments were done for 2nd and 3rd chambers. The number of deformed GUVs and pore formed GUVs were calculated among all the examined GUVs from several images in each time. We calculated the *Fr*_d_ and *Fr*_p_ at different times for an independent experiment. Pore-formed GUVs mean GUVs that have formed pores in their membranes by NPs. The same procedure was performed for 3 independent experiments, and the values of *Fr*_d_ and *Fr*_p_ for each independent experiment were obtained. The sugar concentration-dependent average value with standard deviation of *Fr*_d_ and *Fr*_p_ were calculated at each defined time.

### 2.6 NPs-induced leakage of encapsulating calcein of GUVs

To investigate the NPs-induced leakage of encapsulating calcein of GUVs, 100 μL NPs were added to calcein encapsulated GUVs suspension of 200 μL in a microchamber. We focused on a ‘single GUV’ during interaction. The GUV was recorded using a CCD camera connected with fluorescence mode in a microscope. The starting time of pore formation in the membranes of GUVs occurred when the leakage of calcein started to decrease rapidly. The time-dependent leakage of several GUVs was observed for various sugar concentrations.

## 3. Results

### 3.1 Deformation and compactness of 40%DOPG/60%DOPC-GUVs and DOPC-GUVs induced by 3.33 μg/mL NPs in presence of various sugar concentrations

**Fig 1:**
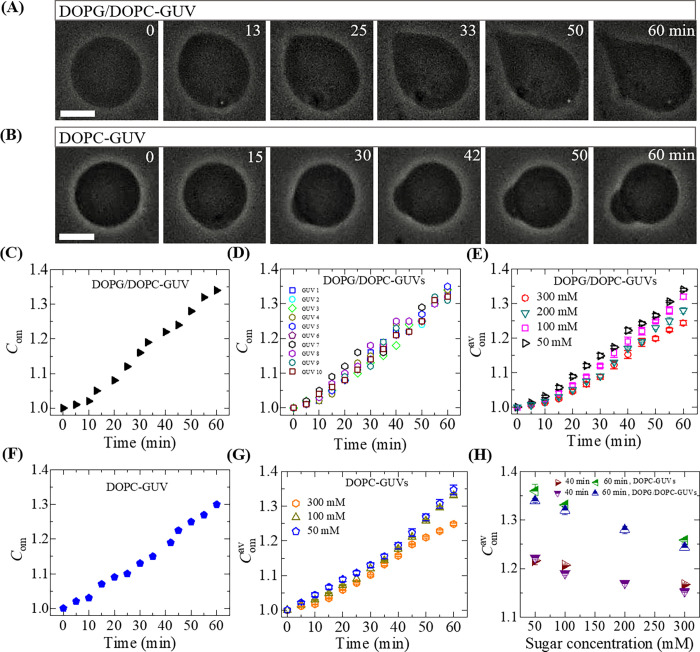
The deformation and compactness of charged and neutral GUVs induced by 3.33 μg/mL NPs. Phase contrast images show the deformation of a (A) 40%DOPG/60%DOPC-GUV and (B) DOPC-GUV in the presence of 50 mM sugar concentration. The numbers on each image indicate the time in minute after addition of NPs. Scale bar is 15 μm. The time course of the change in *C*_*om*_ of (C) 40%DOPG/60%DOPC-GUV and (F) DOPC-GUV as shown in (A) and (B), respectively. (D) The time course of the change in *C*_*om*_ of 10 ‘single 40%DOPG/60%DOPC-GUVs’ in an independent experiment at 50 mM sugar concentration. The time course of average compactness ($C_{om}^{\mathrm{av}}$) of many (E) 40%DOPG/60%DOPC-GUVs and (G) DOPC-GUVs at *c* = 50, 100, 200 and 300 mM. (H) The sugar concentration-dependent $C_{om}^{\mathrm{av}}$ at different times for 40%DOPG/60%DOPC and DOPC-GUVs. Average values with standard deviations of *C*_*om*_ were determined from 3 independent experiments using 15−18 GUVs in each independent experiment.

At first, we investigated the magnetite NPs-induced deformation of 40%DOPG/60%DOPC-GUVs and DOPC-GUVs induced by 3.33 μg/mL NPs. To measure the degree of deformation of a GUV, the corresponding compactness (*C*_*om*_) was determined. The phase contrast images of the dynamic change of a 40%DOPG/60%DOPC-GUV due to the interaction of 3.33 μg/mL NPs under 50 mM sugar concentration is presented in Fig 1(A). The similar experimental results for DOPC-GUVs under the same condition is presented in Fig 1(B). Before addition of NPs into the suspension of GUVs at 50 mM sugar concentration, the vesicles were perfectly spherical in shape as shown in Fig 1(A) and 1(B) at 0 min. As the time progressed, the vesicles started to deform from their spherical shape, and the deformation was observed to increase with time. At 0 min (before interacting the NPs), the value of *C*_*om*_ was 1.0 for both GUVs, and it increased with time after interacting with NPs. In Fig 1(A), the values of *C*_*om*_ were 1.05, 1.12, 1.19, 1.28, and 1.34 at 13, 25, 33, 50 and 60 min, respectively. For neutral GUVs, the values of *C*_*om*_ were 1.07, 1.13, 1.20, 1.25 and 1.30 at 15, 30, 42, 50 and 60 min, respectively as shown in Fig 1(B). The time-dependent change of *C*_*om*_ of Fig 1(A) and 1(B) are presented in Fig 1(C) and 1(F), respectively. The *C*_*om*_ increases with time for both GUVs. These deformations were similar to those observed before in our experiment for a fixed sugar concentration [30]. Similar experiments were conducted for many GUVs (number of measured GUVs, *N* = 15) to confirm the reproducibility. To compare the compactness of different GUVs, the time course of the change in *C*_om_ of 10 ‘single 40%DOPG/60%DOPC-GUVs’ in an independent experiment at 50 mM sugar concentration is shown in Fig 1(D). These results clearly indicate that when NPs are adsorbed into the membrane of GUVs, the vesicles became deformed. The deformation of GUVs for three other sugar concentrations was also investigated, namely, *c* = 100, 200 and 300 mM, and then the corresponding *C*_*om*_ values were measured.

It is necessary to explain how to calculate the average compactness, $C_{om}^{\mathrm{av}}$. For example, in the first experiment, the value of mean compactness at time 30 min was 1.150 for 15 different GUVs (*N* = 15) under *c* = 50 mM. The values of mean compactness at time 30 min were 1.142 for *N* = 17 and 1.149 for *N* = 18, respectively in the second and third experiments under the same sugar concentration. Therefore, the $C_{om}^{\mathrm{av}}$ at 30 min for 3 independent experiments (number of independent experiment, *n* = 3) was (1.150 + 1.142 + 1.149)/3 using *N* = 15–18 under *c* = 50 mM. $C_{om}^{\mathrm{av}}$ for other times were also measured under the same conditions.

The change in $C_{om}^{\mathrm{av}}$ with time for different sugar concentrations is shown in Fig 1(E) for charged 40%DOPG/60%DOPC-GUVs and in Fig 1(G) for neutral DOPC-GUVs. In Fig 1(E) and 1(G), it is clearly observed that the rate of increment of $C_{om}^{\mathrm{av}}$ with time is different for different sugar conditions. We also calculated the sugar concentration-dependent $C_{om}^{\mathrm{av}}$. Fig 1(H) shows the sugar concentration-dependent $C_{om}^{\mathrm{av}}$ for both charged and neutral GUVs, respectively, in which the $C_{om}^{\mathrm{av}}$ decreased with the increase of sugar concentration. As for example, the value of $C_{om}^{\mathrm{av}}$ was (1.22 ± 0.01) for *c* = 50 mM and (1.15 ± 0.01) for *c* = 300 mM at 40 min for 40%DOPG/60%DOPC-GUVs. Additionally, the value of $C_{om}^{\mathrm{av}}$ was (1.35 ± 0.01) for *c* = 50 mM and (1.25 ± 0.01) for *c* = 300 mM at 60 min for DOPC-GUVs. These investigations suggested that higher concentrations of sugar inhibited the deformation of lipid vesicles.

### 3.2 Fraction of deformed 40%DOPG/60%DOPC-GUVs induced by 3.33 μg/mL NPs under various sugar concentrations

**Fig 2:**
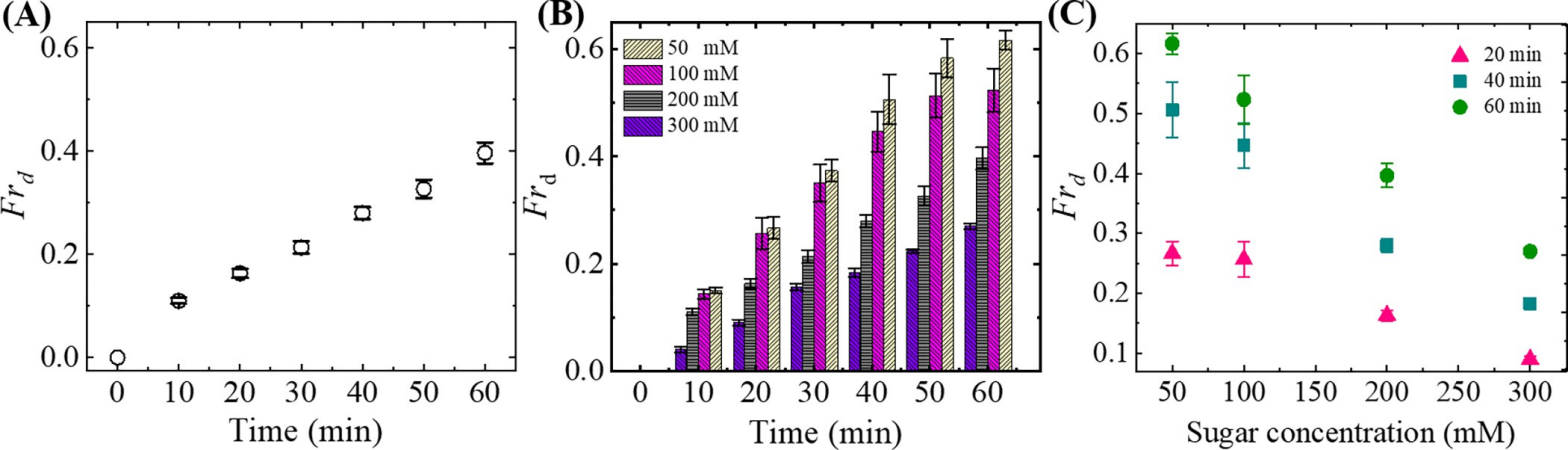
Fraction of deformed 40%DOPG/60%DOPC-GUVs at 3.33 μg/mL. (A) The time course of average *Fr*_d_ at 200 mM sugar concentration. (B) Bar chart of *Fr*_d_ with time for various sugar concentrations. (C) The sugar concentration-dependent *Fr*_d_ at 20, 40 and 60 min. The average values with standard deviations (in B, C, D) were obtained from 3 independent experiments containing 15–20 GUVs in each independent experiment.

In section 3.1, we studied the NPs-induced deformation of an individual GUV and their corresponding *C*_*om*_. Now, we describe the interaction of 3.33 μg/mL NPs on the ensemble of 40%DOPG/60%DOPC-GUVs under various concentrations of sugar. Addition of NPs into the suspension of vesicles deformed some GUVs, pore formed some GUVs, and some GUVs remain unchanged. Let us explain how to calculate the fraction of deformed GUVs (*Fr*_d_) at different time. In this regard, after addition of NPs into the GUVs suspension in the microchamber, images of GUVs were taken at 0, 10, 20, 30, 40, 50, and 60 min without changing the microscope focusing position. Similar experiments were done for second and third chamber. Next, we counted the number of deformed GUVs among all the examined GUVs (*N* = 15−20) from several phase contrast images at time 0, 10, 20, 30, 40, 50 and 60, min. Deformed GUVs mean GUVs that have deformed by NPs. In the first independent experiment, if 50 GUVs were counted from several images from where 10 GUVs were deformed at 20 min after mixing the NPs, the value of *Fr*_d_ = 0.20 at that time. We calculated the *Fr*_d_ for different times. We considered these investigations as independent experiments. The values of *Fr*_d_ for 40%DOPG/60%DOPC-GUVs at time 0, 10, 20, 30, 40, 50, and 60 min were obtained 0, 0.10, 0.18, 0.22, 0.30, 0.32, and 0.40, respectively, under *c* = 200 mM. These data confirmed that *Fr*_d_ increased with time. We followed the similar procedure as described above for 3 times (*n* = 3). The average value with standard deviation of *Fr*_d_ obtained from those independent experiments for *c* = 200 mM with time is shown in Fig 2(A). The average values of *Fr*_d_ at 0, 10, 20, 30, 40, 50 and 60 min were obtained 0, (0.11 ± 0.01), (0.16 ± 0.01), (0.21 ± 0.01), (0.28 ± 0.01), (0.33 ± 0.02) and (0.40 ± 0.02), respectively (Fig 2A). The average value of *Fr*_d_ also increased with time. We carried out the similar type of experiments for a variety of sugar concentrations, such as 50, 100, 200 and 300 mM. The time-dependent bar diagram for the average *Fr*_d_ at 3.33 μg/mL under various sugar concentrations is shown in Fig 2(B). At 40 min, the values of *Fr*_d_ obtained were (0.51 ± 0.05), (0.45 ± 0.04), (0.28 ± 0.01) and (0.18 ± 0.01) for *c* = 50, 100, 200 and 300 mM, respectively. So, the average *Fr*_d_ decreased with the increase of sugar concentration. This result is clearly depicted in Fig 2(C) where the sugar concentration-dependent average *Fr*_d_ at different intervals of time is shown. Therefore, as the sugar concentration in buffer solution increased, the *Fr*_d_ decrease, indicating the inhibition of deformation of vesicles with sugar concentration.

### 3.3 Fraction of deformed 40%DOPG/60%DOPC-GUVs under various NPs concentrations at 50 mM sugar

**Fig 3:**
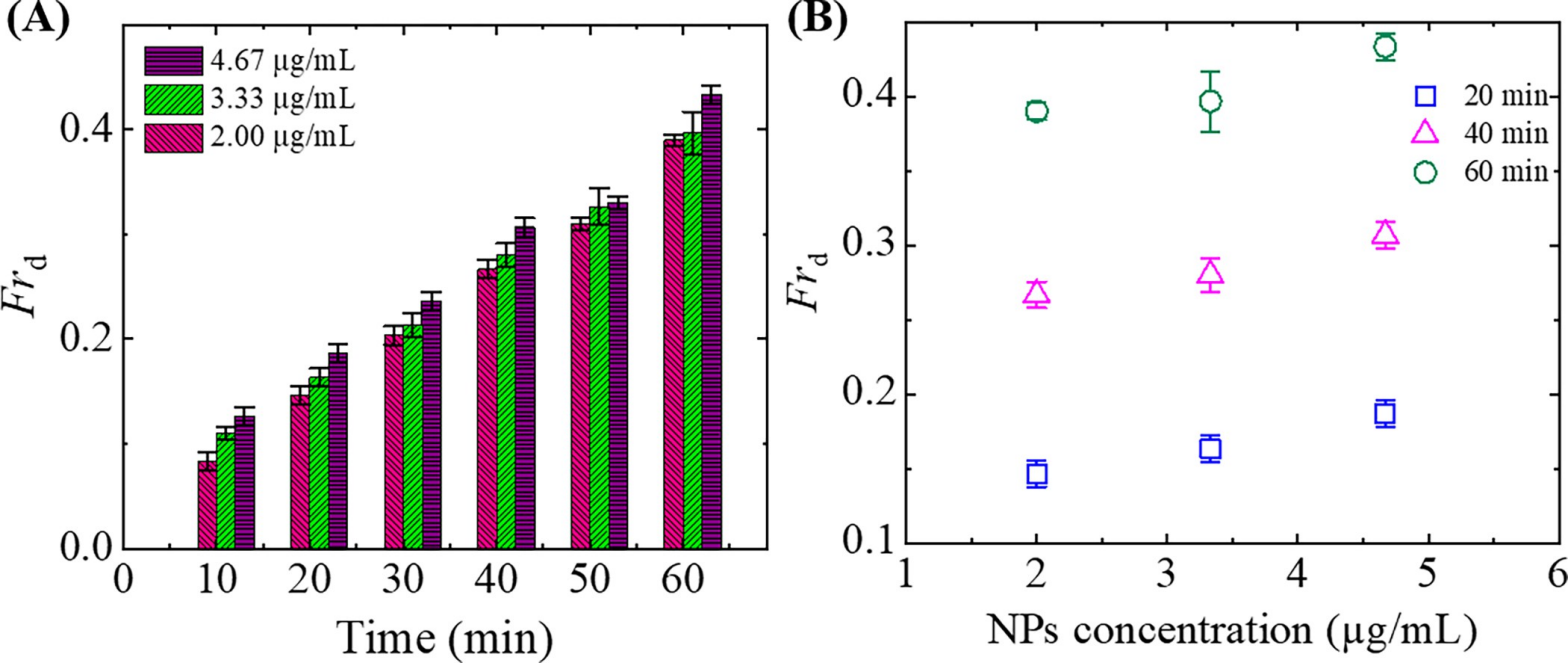
Fraction of deformed 40%DOPG/60%DOPC-GUVs under various NPs concentrations at 50 mM sugar. (A) The bar diagram of *Fr*_d_ for 2.00, 3.33 and 4.67 μg/mL NPs. (B) The linear proportionality between NPs concentration and *Fr*_d_ at different times. The average values with standard deviations were obtained from 3 independent experiments containing 15–20 GUVs in each independent experiment.

It is essential to investigate *Fr*_d_ at various NPs concentrations, keeping the sugar concentration constant. Here, we present the results from the study of deformation of 40%DOPG/60%DOPC-GUVs under 2.00, 3.33 and 4.67 μg/mL NPs concentrations in GUVs suspension at 50 mM sugar concentration. The bar diagram of time-dependent average *Fr*_d_ under various concentrations of NPs is shown in Fig 3(A). The *Fr*_d_ increases with time for all concentrations. The NPs concentration-dependent average *Fr*_d_ at 20, 40 and 60 min is shown in Fig 3(B). These results clearly indicate that *Fr*_d_ increases with increasing NPs concentration in the GUVs suspension. For example, the value of *Fr*_d_ at 40 min was (0.27 ± 0.01), (0.28 ± 0.01) and (0.31 ± 0.01) for 2.00, 3.33 and 4.67 μg/mL NPs concentration, respectively. A similar trend to increase the fraction of deformed GUVs was also obtained for other times.

### 3.4 NPs-induced leakage of calcein from the inside of 40%DOPG/60%DOPC-GUVs and DOPC-GUVs

**Fig 4:**
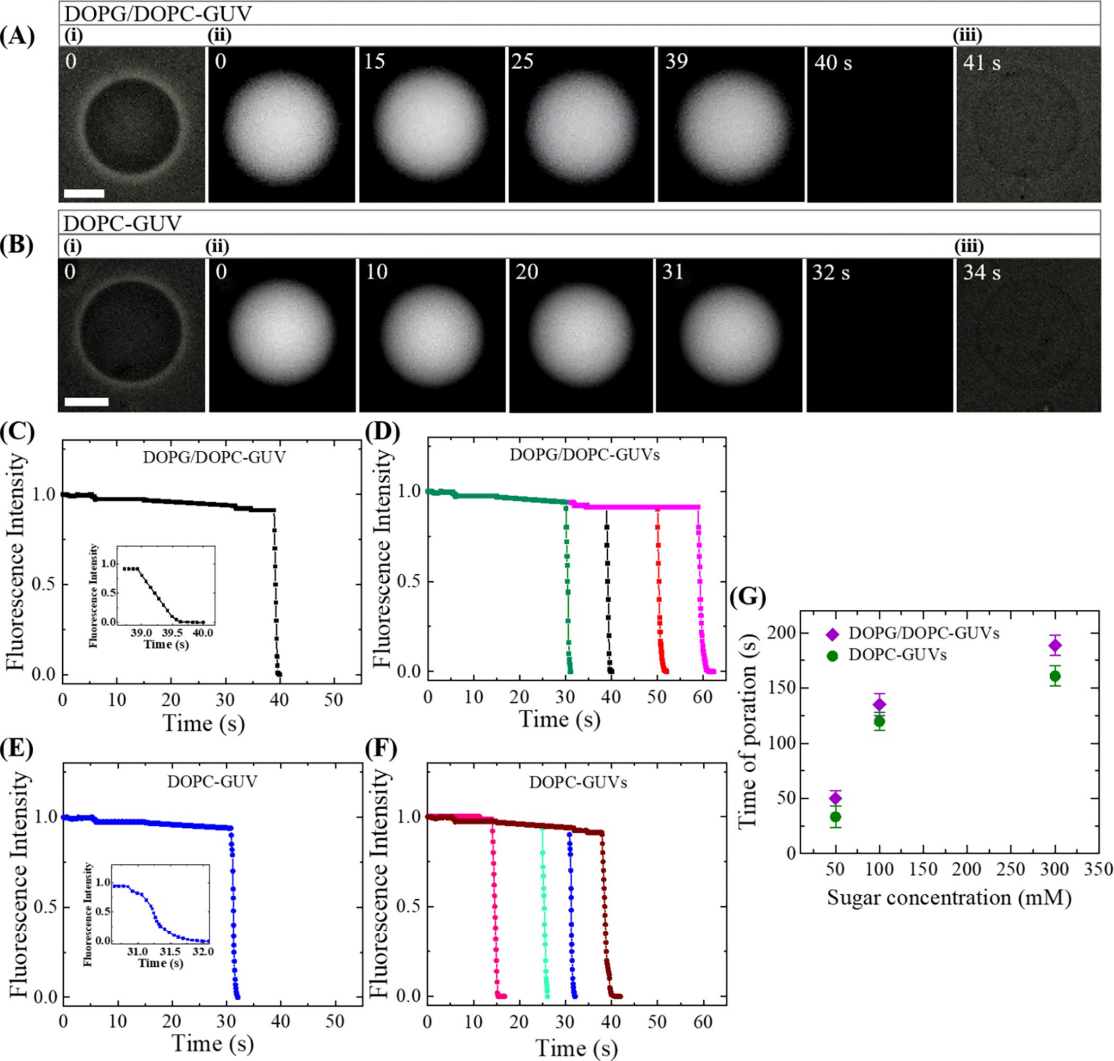
Pore formation in the membranes of GUVs induced by 3.33 μg/mL NPs. Fluorescence images of (A) 40%DOPG/60%DOPC-GUVs and (B) DOPC-GUVs show the progressive decrease of calcein from inside the GUVs at 50 mM sugar concentration. The numbers on each image show the time in seconds after the NPs are added in the suspension of GUVs. The bar corresponds to 15 μm. Time course of the change of normalized fluorescence intensity of calcein for (C) 40%DOPG/60%DOPC-GUVs and (E) DOPC-GUVs as shown in (A) and (B), respectively. (D, F) Under the same condition corresponding to (C) and (E), the change in normalized fluorescence intensity with time for many GUVs under 50 mM sugar concentration. (G) Sugar concentration-dependent average time of pore formation in 40%DOPG/60%DOPC and DOPC-GUVs.

We investigated the lipid membrane poration of 40%DOPG/60%DOPC-GUVs and DOPC-GUVs induced by 3.33 μg/mL NPs under various concentration of sugar by observing the leakage of fluorescent probe (calcein) from the inside of GUVs. It is to be mentioned here that the Stokes-Einstein radius (*R*_SE_) of calcein is 0.74 nm [68]. The dynamic change of a GUV is investigated in Fig 4(A) for 40%DOPG/60%DOPC-GUVs and in Fig 4(B) for DOPC-GUVs due to the interaction of 3.33 μg/mL NPs under 50 mM sugar concentration. Before interacting NPs with the surface of GUVs, it had a high contrast in phase contrast image at 0 s as shown in Fig 4A(i) and 4B(i) due to the difference of refractive index between the internal (sucrose) and external (glucose) solutions of GUVs. The corresponding GUVs in fluorescence microscopy are shown in Fig 4A(ii) and 4B(ii), which showed high concentration of fluorescent material in the inside of vesicles at this time. During interaction of NPs with GUVs, the fluorescence intensity was almost same until time 39 s (Fig 4A(ii)) and 31 s (Fig 4B(ii)), and then the intensity decreased rapidly with time. The fluorescence intensity became zero at 40 s and 32 s of the corresponding GUVs. After the complete leakage of calcein, the phase contrast images of the corresponding GUVs were spherical and intact with undetectable break as shown in Fig 4A(iii) and 4B(iii). The calcein leakage occurred due to the formation of pores in the membranes induced by NPs. The similar type of the change in fluorescence intensity was also observed for the peptide-induced pore formation in lipid membranes of vesicles [44]. The size of such pores was so small that the vesicles were intact with spherical structure after ejecting the internal calcein. The time-dependent normalized fluorescence intensity of GUVs as shown in Fig 4A and 4B are presented in Fig 4C and 4E, respectively. The intensity was almost same until 39 s (Fig 4C) and 31 s (Fig 4E), followed by a sharp decrease to zero. The sharp decrements occurred within one second and this transformation can be viewed more precisely from the insets of Fig 4C and 4E. The moment of time at which the fluorescence intensity began to rapidly decrease is defined as the time of NPs-induced pore formation in the membranes of GUVs. The fluorescence intensity decreases due to the leakage of calcein from the inside of GUVs to outside. Therefore, there were two states of GUVs due to the interaction of NPs; one was intact state in which the fluorescence intensity was same over time and the another was the pore state in which the fluorescence intensity was decreased rapidly over time and reached to zero. When we conducted similar experiments using many GUVs (*n* = 15–20) such type of transition from intact state to pore state was also observed. In Fig 4D and 4F, we presented the time-dependent fluorescence intensity of several 40%DOPG/60%DOPC-GUVs and DOPC-GUVs, respectively, under 50 mM sugar concentration. The time of pore formation for several GUVs was not same under the same condition. Thus, the nature of pore formation was stochastic. The reproducibility was checked in several independent experiments using many GUVs. Similar experiments were also done for many GUVs under 100 and 300 mM sugar concentration at 3.33 μg/mL NPs. In those conditions, the pore formation was also in stochastic nature.

The average time of pore formation in stochastic nature under 300 mM sugar concentration was higher than that of 50 and 100 mM sugar concentrations. Fig 4G show the sugar concentration-dependent average time of poration in 40%DOPG/60%DOPC and DOPC-GUVs. In both charged and neutral membranes, the average time for pore formation increased with the increase of sugar concentration in the solution. These investigations clearly indicated that higher sugar concentrations inhibited the pore formation in vesicles.

### 3.5 Fraction of deformed and pore formed 40%DOPG/60%DOPC and DOPC-GUVs

**Fig 5:**
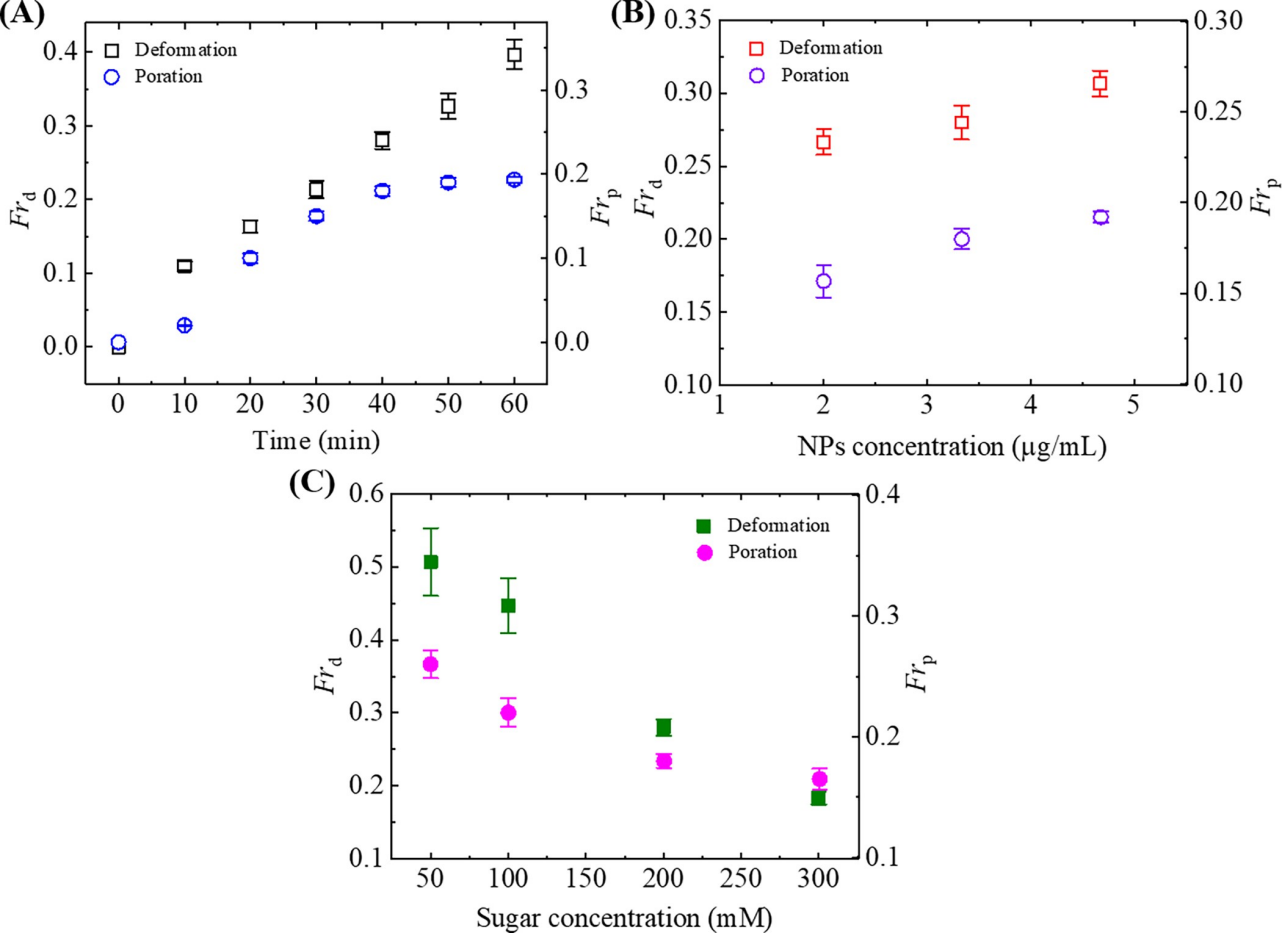
Fraction of deformed and pore formed 40%DOPG/60%DOPC-GUVs. (A) The time-dependent *Fr*_d_ and *Fr*_p_ under 3.33 μg/mL NPs at 200 mM sugar concentration. (B) The NPs concentration-dependent *Fr*_d_ and *Fr*_p_. (C) The sugar concentration-dependent *Fr*_d_ and *Fr*_p_ using 3.33 μg/mL NPs at 40 min. The average values with standard deviations were obtained from 3 independent experiments containing 15–20 GUVs in each independent experiment.

So far, we have observed two types of phenomena for the interaction of NPs with GUVs; one was deformation, and another was poration. It means that some GUVs became deformed and some GUVs formed pores after interacting NPs. Therefore, it is very important to represent the results using a single graph. The fraction of pore formed GUVs is defined by *Fr*_p_. The time-dependence of both *Fr*_d_ and *Fr*_p_ is presented in Fig 5A, in which GUVs was interacted with 3.33 μg/mL NPs under 200 mM sugar concentration. While the value of *Fr*_d_ increased with time, but the value of *Fr*_p_ at first increased with time and then remained steady over time. Hence, the difference in *Fr*_d_ and *Fr*_p_ until time 20 min was not significant as shown in Fig 5A. This difference increased with time. The maximum difference was obtained at time 50 to 60 min. We repeated the experiment 3 times using many GUVs and obtained the average fractions with standard deviations as shown in Fig 5. Similar experiments for various NPs concentrations under the same sugar concentration were done and those fractions were measured. The NPs concentration-dependent *Fr*_d_ and *Fr*_p_ are presented in Fig 5(B). Both the fractions increased with the increase of NPs concentration under 200 mM sugar concentration. However, the rate of increase of those fractions is different. The rate of increase of *Fr*_d_ was much higher that of *Fr*_p_ at different NPs concentrations. For example, *Fr*_d_ was 0.31 ± 0.01 and *Fr*_p_ was 0.19 ± 0.01 at 4.67 μg/mL NPs. Hence, the tendency of deformation was higher than the tendency of poration under the same sugar concentration. We performed similar investigations under various concentrations of sugar in buffer solution. Fig 5C shows the sugar concentration-dependent *Fr*_d_ and *Fr*_p_ using 3.33 μg/mL NPs at 40 min. Both the fractions decreased with sugar concentration. The rate of decrement of *Fr*_d_ was slightly higher than the rate of decrement of *Fr*_p_. Hence, the increased sugar concentration in buffer solution reduced these fractions.

**Fig 6:**
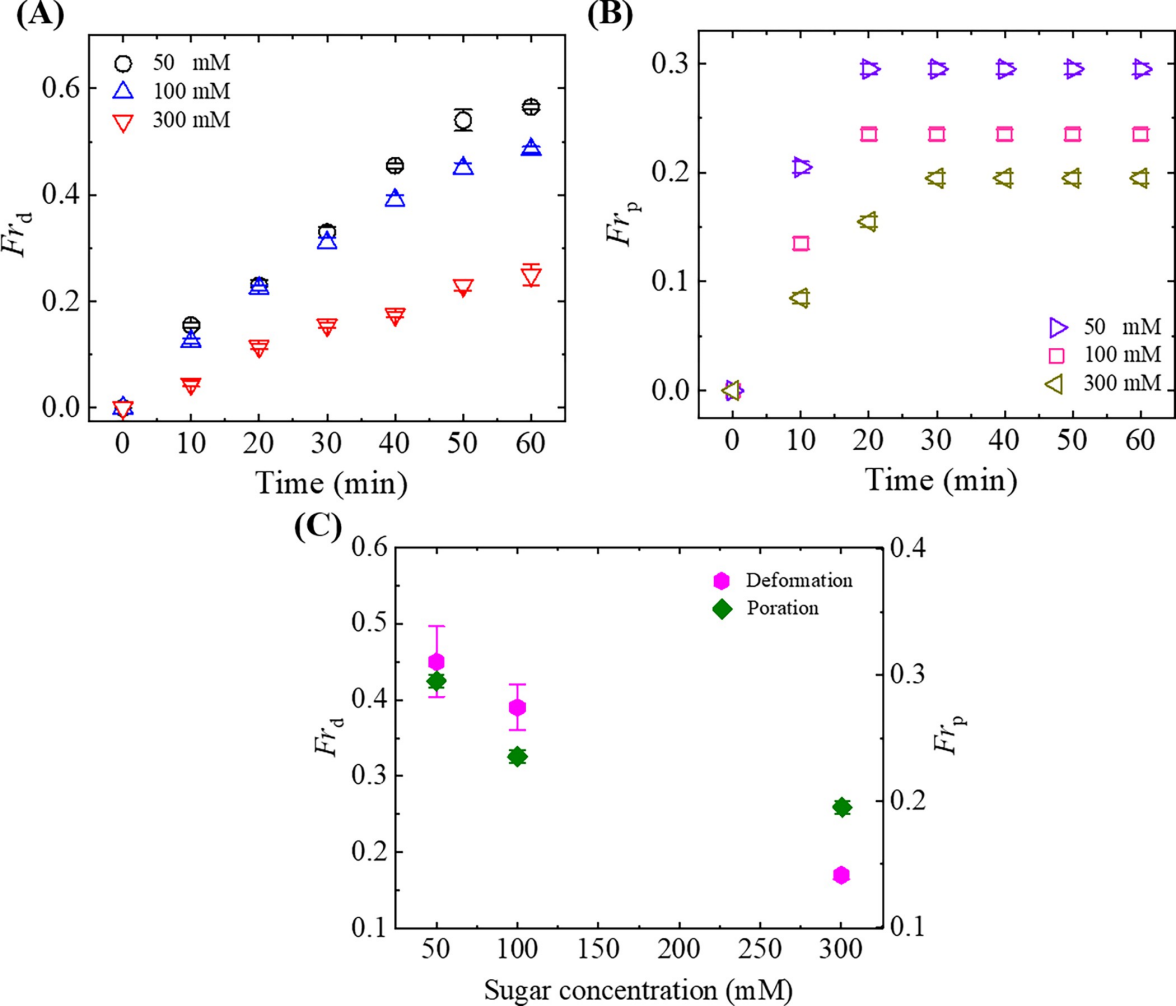
Fraction of deformed and pore formed DOPC-GUVs. The time-dependent (A) *Fr*_d_ and (B) *Fr*_p_ under 3.33 μg/mL NPs at *c* = 50, 100 and 300 mM. (C) The sugar concentration-dependent *Fr*_d_ and *Fr*_p_ using 3.33 μg/mL NPs at 40 min. The average values with standard deviations were obtained from 3 independent experiments containing 15–20 GUVs in each independent experiment.

To understand the trend of *Fr*_d_ and *Fr*_p_, we also calculated those fractions in case of neutral DOPC-GUVs. The time course of *Fr*_d_ is depicted in Fig 6A while Fig 6B presents the time course of *Fr*_p_ under various sugar concentrations, i.e., *c* = 50, 100 and 300 mM. Here, both of those fractions also increased with time for each sugar concentration and *Fr*_p_ remained constant from 30 s (Fig 6B). At a certain time, both the *Fr*_d_ and *Fr*_p_ are higher at lower sugar concentration. For instance, at 50 min, *Fr*_d_ were (0.54 ± 0.02), (0.45 ± 0.01) and (0.23 ± 0.01) for *c* = 50, 100 and 300 mM, respectively, as shown in Fig 6A. On the other hand, *Fr*_p_ were (0.30 ± 0.01), (0.24 ± 0.01) and (0.20 ± 0.01) for *c* = 50, 100 and 300 mM, respectively, at 50 min as shown in Fig 6B. The decrease in *Fr*_d_ and *Fr*_p_ is evident from Fig 6C where the sugar concentration-dependent *Fr*_d_ and *Fr*_p_ using 3.33 μg/mL NPs at 40 min is presented. Therefore, it can be said that increase in sugar concentration decreased the deformation and pore formation of both charged and neutral GUVs.

### 3.6 Comparison of fraction of deformation and poration for 40%DOPG/60%DOPC-GUVs and DOPC-GUVs under various concentrations of sugar

**Fig 7:**
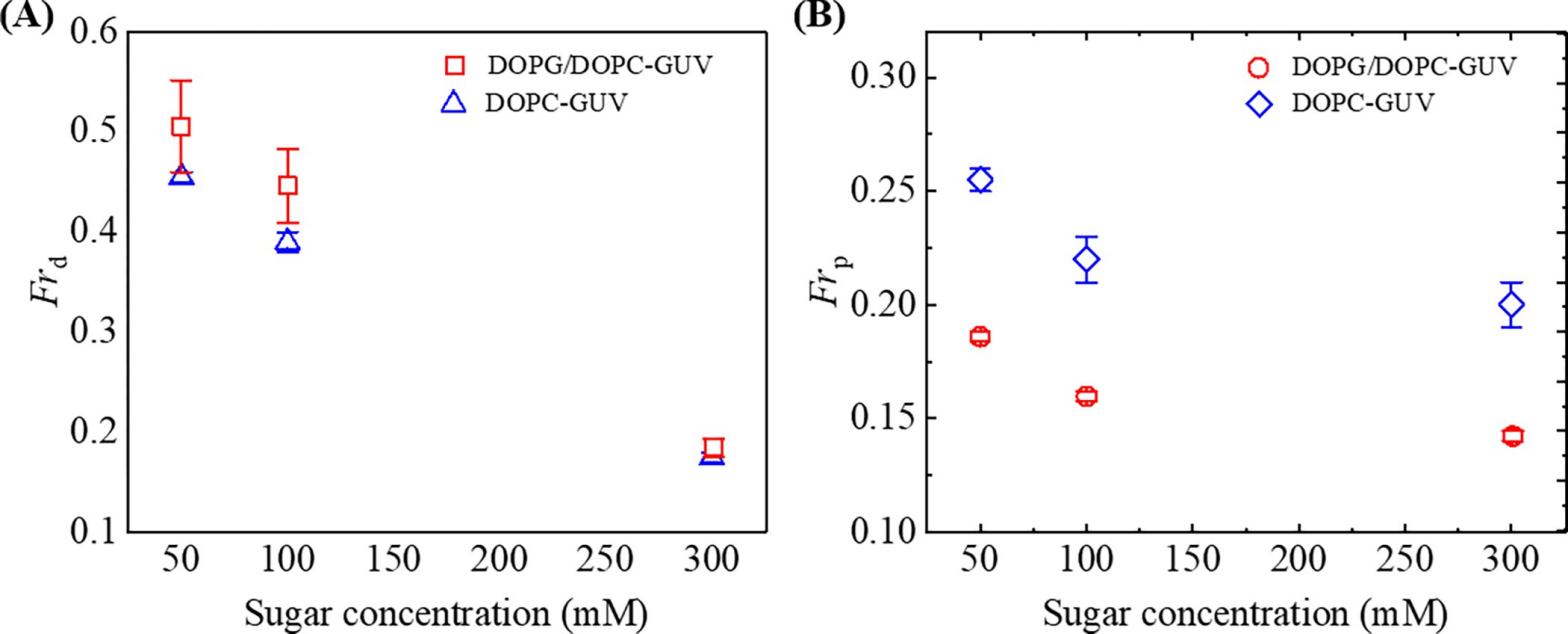
Comparison between the fraction of deformed and pore formed 40%DOPG/60%DOPC-GUVs and DOPC-GUVs under various sugar concentration at 3.33 μg/mL NPs. (A) The sugar concentration-dependent *Fr*_d_ for 40%DOPG/60%DOPC-GUVs and DOPC-GUVs at 40 min. (B) The sugar concentration-dependent *Fr*_p_ for 40%DOPG/60%DOPC-GUVs and DOPC-GUVs at 40 min. The average values with standard deviations were obtained from 3 independent experiments containing 15–20 GUVs in each independent experiment.

In this section, the comparison between the *Fr*_d_ and *Fr*_p_ of charged 40%DOPG/60%DOPC-GUVs and neutral DOPC-GUVs under different sugar concentrations is described. Fig 7 shows this comparison very well. For example, at *c* = 100 mM, *Fr*_d_ were (0.45 ± 0.04) and (0.39 ± 0.01), for charged and neutral GUVs, respectively (Fig 7A), and under the same sugar concentration, *Fr*_p_ were (0.24 ± 0.01) and (0.22 ± 0.01) for charged and neutral GUVs, respectively (Fig 7B). Both the *Fr*_d_ and *Fr*_p_ decreased with sugar concentration. The value of *Fr*_d_ is higher in charged membrane than the neutral ones, whereas the value of *Fr*_p_ is in opposite view. Table 1 depicts the values of *Fr*_d_ and *Fr*_p_. From these findings the statement is clear that the probability of deformation is higher for charged membranes compared to neutral ones, whereas the probability of pore formation shows the opposite nature at a fixed sugar condition.

**Table 1:** Fraction of deformation and poration in 40%DOPG/60%DOPC-GUVs and DOPC-GUVs for 3.33 μg/mL NPs at 40 min.

Sugar conc. (mM)	Fraction of deformation		Fraction of poration	
	DOPG/DOPC-GUVs	DOPC-GUVs	DOPG/DOPC-GUVs	DOPC-GUVs
50	0.51 ± 0.05	0.45 ± 0.05	0.26 ± 0.01	0.30 ± 0.01
100	0.45 ± 0.04	0.39 ± 0.03	0.22 ± 0.01	0.24 ± 0.01
200	0.28 ± 0.01	-	0.18 ± 0.01	-
300	0.18 ± 0.01	0.17 ± 0.01	0.17 ± 0.01	0.20 ± 0.01

### 3.7 Different types of deformation of charged and neutral GUVs due to the interaction of 3.33 μg/mL NPs

**Fig 8:**
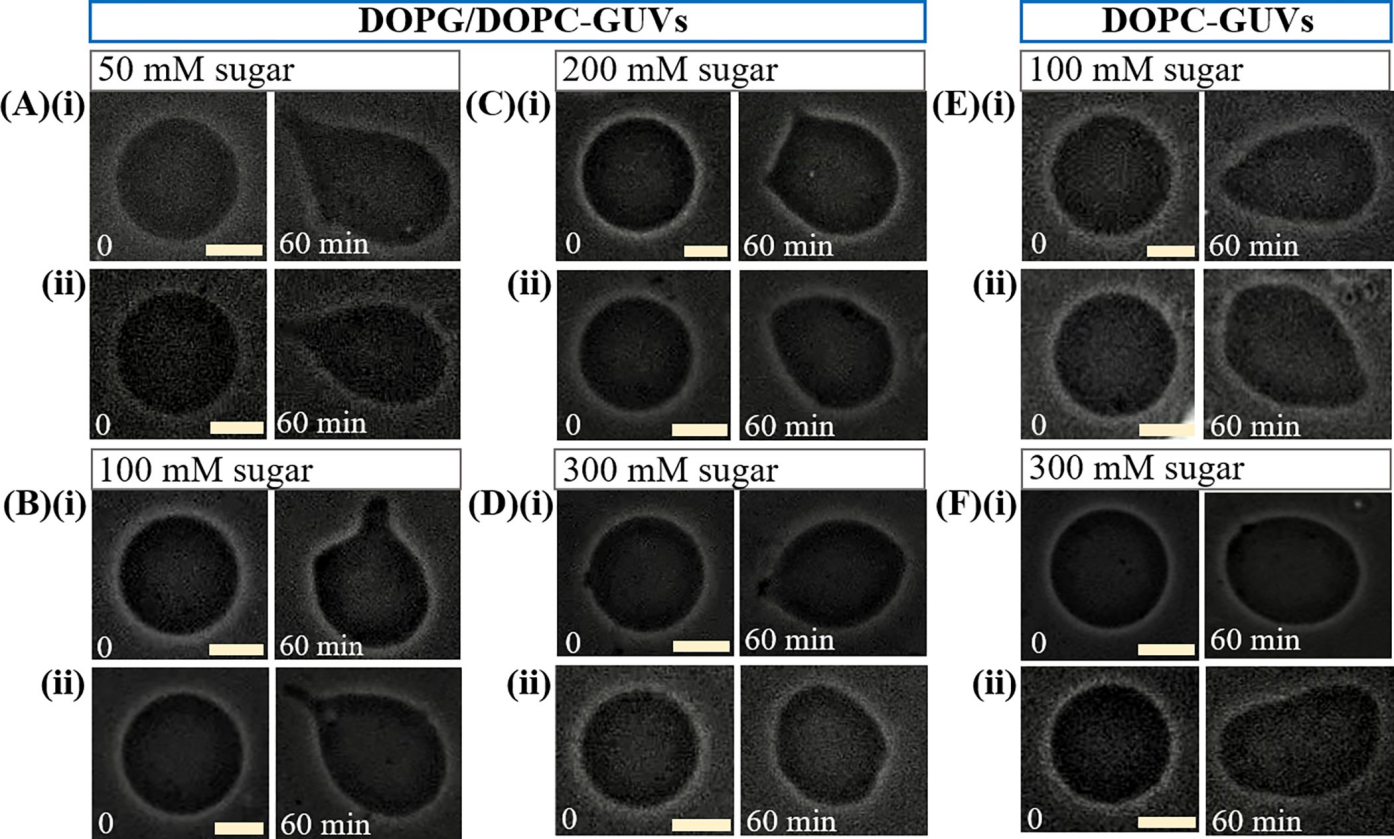
Different types of deformation of charged and neutral GUVs due to the interaction of 3.33 μg/mL NPs under (A) 50 (B) 100 (C) 200 and (D) 300 mM sugar concentrations. The ‘common murre egg-shaped’ 40%DOPG/60%DOPC-GUVs in A(i, ii)) and B(ii); ‘winnoing fan-shaped’ 40%DOPG/60%DOPC-GUVs in B(i) and C(i); ‘oval-shaped’ 40%DOPG/60%DOPC-GUVs in C(ii) and D(i, ii); ‘common murre egg-shaped’ DOPC-GUVs in E(i, ii)), and ‘Oval-shaped’ DOPC-GUVs in F(i, ii). The bar corresponds to 15 μm.

In section 3.1, we presented the deformation of 40%DOPG/60%DOPC and DOPC-GUVs due to the interaction of 3.33 μg/mL NPs under various sugar concentration. But in our experiments, we observed different sorts of deformations. Various types of deformation of 40%DOPG/60%DOPC and DOPC-GUVs due to the interaction of 3.33 μg/mL NPs are shown in Fig 8 at 50, 100, 200 and 300 mM sugar concentrations. In all conditions, the GUVs were spherical shape at 0 min (i.e., before interacting NPs with GUVs). After interaction of NPs with membranes, the final shape of GUVs is shown at 60 min. The ‘common murre egg-shaped’ 40%DOPG/60%DOPC-GUVs are observed in Fig 8A(i, ii) and 8B(ii) at 50 and 100 mM sugar, respectively. In Fig 8B(i) and 8C(i), ‘winnowing fan-shaped’ 40%DOPG/60%DOPC-GUVs are observed for 100 and 200 mM sugar concentration, respectively. ‘Oval-shaped’ 40%DOPG/60%DOPC-GUVs are observed in Fig 8C(ii) and 8D(i, ii) at 200 and 300 mM sugar, respectively. In the similar way, the ‘common murre egg-shaped’ DOPC-GUVs are observed in Fig 8E(i, ii), and ‘Oval-shaped’ DOPC-GUVs are observed in 8F(i, ii) at 100 and 300 mM sugar, respectively.

## 4. Discussion

**Fig 9:**
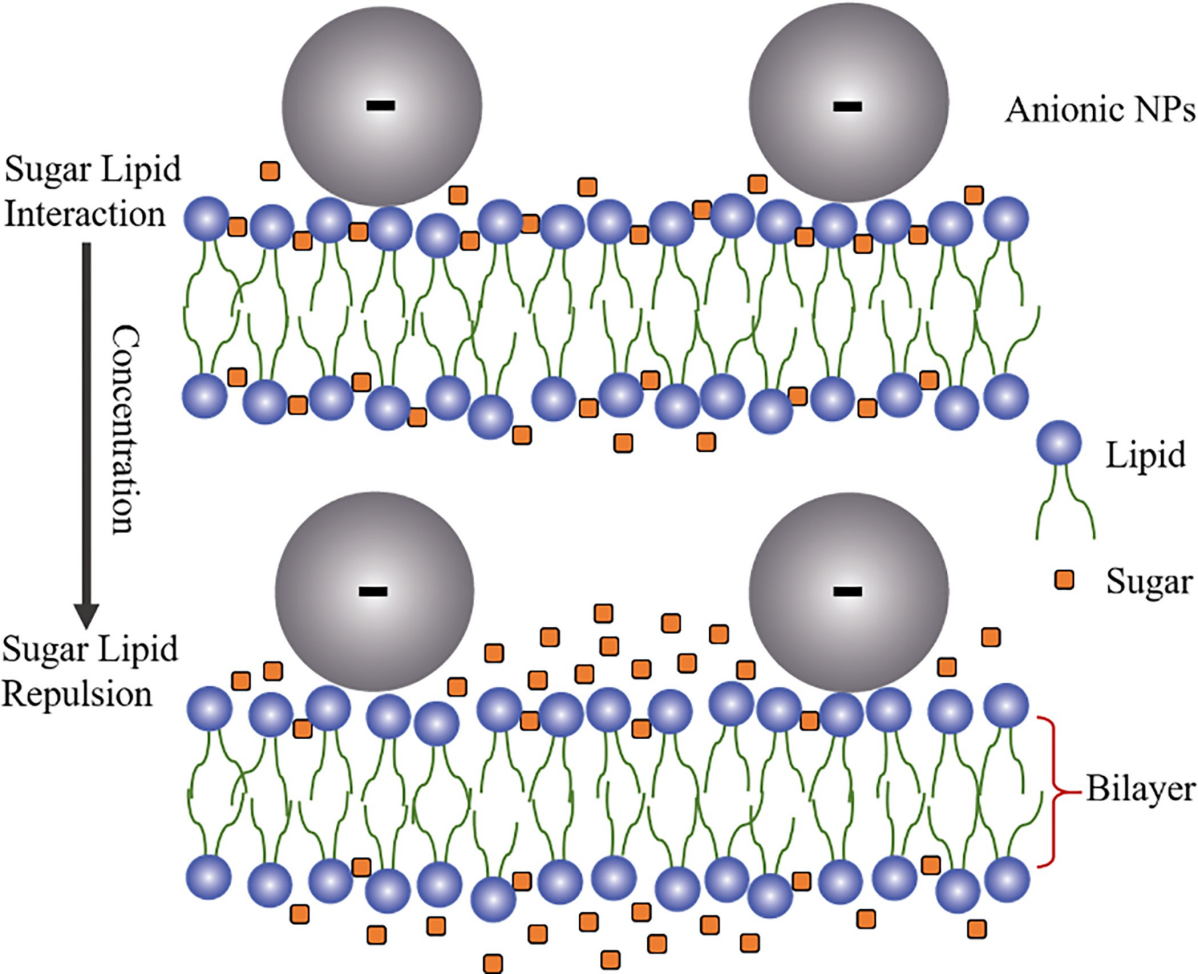
Effects of sugar molecules at lower and higher concentrations in the lipid bilayer for a specific concentration of NPs. The sugar-lipid head groups attractive interaction at lower sugar concentration is attributed to the enthalpy-driven interaction. At higher sugar concentrations, however, the sugar-lipid head groups repulsion is attributed to entropy-driven repulsion. The number density of bound sugar molecules at the interface of lipid molecules in the membrane is higher at lower sugar concentrations than that of higher ones. The sugar molecules at higher sugar concentration are repelled by the lipid molecules.

The interaction of anionic magnetite NPs with 40%DOPG/60%DOPC and DOPC-GUVs was studied under various sugar concentration in aqueous solution. The GUVs became deformed from their spherical shape upon adoption of NPs into the membrane interface as shown in Fig 1(A) and 1(B). The value of compactness increased with time under various sugar concentrations as shown in Fig 1(C)–1(G). The shape of vesicles is determined by the minimization of elastic energy of the closed bilayer membrane. According to the ‘bilayer-coupling model’ [69], the elastic energy of GUVs (*W*_el_) is due to only the bending energy of membranes (*W*_b_) as the model did not consider elastic stretching of the monolayers. Hence, the minimum elastic energy is determined by the area difference between the outer monolayer (*A*^out^) and the inner monolayer (*A*^in^), i.e., Δ*A* = *A*^out^−*A*^in^ for a given area *A*, and a given volume *V* of the GUVs [70–72]. Later, the shape change (i.e., deformation) of vesicles was well explained by the area-difference-elasticity model (ADE model) [73, 74]. In the ADE model, the area of each monolayer is not fixed to the equilibrium area but can stretch elastically to increase the nonlocal elastic energy of the membranes. The equilibrium shapes of vesicles are assumed to correspond to the minimum of elastic energy of closed bilayer membrane. The elastic energy (*W*_el_) is considered as the sum of bending energy of membrane (*W*_b_) and the energy of relative monolayer stretching (*W*_r_) as follows.

$$\begin{array}{l} W_{\mathrm{el}}=W_{b}+W_{r}\\ \;=\frac{1}{2}k_{c}{\int \left(C_{1}+C_{2}\right)}^{2}dA+\frac{1}{2}\frac{k_{r}}{A_{0}h^{2}}{\left(\Delta A-\Delta A_{0}\right)}^{2} \end{array}$$ (2)

where, *k*_c_ is the local bending modulus and *k*_r_ is the nonlocal bending modulus of membrane. The principal curvatures are *C*_1_, and *C*_2_. Integration is performed over the neutral surface of the bilayer with area *A*_0_. The area difference between the outer and the inner monolayer of the bilayer is denoted by Δ*A*. The distance between the neutral surfaces of two monolayers is *h*. The area difference between the two monolayers in the GUVs at equilibrium (i.e., unstretched monolayers) is $\Delta A_{0}=A_{o}^{\mathrm{out}}-A_{o}^{\mathrm{in}}$. The difference in area between the external monolayer and the internal monolayer of membranes is Δ*A* = *A*^out^−*A*^in^ at stretched state. According to ADE model, the shape of GUVs is determined by the minimization of *W*_el_ for a given area *A*, a given volume *V*, and a given equilibrium (i.e., relaxed) area difference Δ*A*_0_. Under constant volume of GUVs, the deformation of GUVs from spherical to prolate/any other shape is due to (Δ*A*−Δ*A*_0_)^2^. As the value of this difference increased with time, the deformation (i.e., compactness) of vesicles increased as shown in Fig 1(C)–1(G). The ADE model can also explain the deformation of GUVs with various types of shape as shown in Fig 8. As the sugar concentration increased, the value of average compactness decreased as shown in Fig 1(H). It is reasonably considered that the presence of higher concentration of sugar reduces the mismatch of area between the two layers, induces the decrease of degree of deformation as shown in Fig 1(H). This description is also valid in explaining the results of fraction of deformed GUVs with time and also with sugar concentration as shown in Fig 2. As the NPs concentration increased, the surface pressure in the outer monolayer increased which mismatches more area between the outer monolayer and inner monolayer. The enhanced mismatch area deformed more fraction of GUVs with the increase of NPs concentration as shown in Fig 3. Based on the above discussion, the interaction of NPs with lipid membrane under the influence of sugar concentration is illustrated in Fig 9. We used 50 mM sugar for lower concentration and 300 mM sugar for higher concentration. At lower sugar concentration, the sugar-lipid head groups attractive interaction is attributed to the enthalpy-driven interaction. At higher sugar concentration, sugar-lipid head groups repulsion is attributed to the entropy-driven repulsion. These attractive and repulsive nature of sugar with lipid molecules is also reported by other groups [50, 51]. At a specific NPs concentration, the surface pressure at higher sugar concentrations is lower compared to lower sugar concentrations. The surface pressure changes because of the lower/higher number of bound sugar molecules at the membrane interface at a specific concentration of NPs. The differences in surface pressure at lower/higher sugar concentrations influence the stability of the lipid bilayer. As the surface pressure at lower sugar concentration is higher compared to higher sugar concentration, therefore, the compactness and the possibility of deformation decreased with the increase of sugar concentration (Fig 1H and 2C).

Now we explain the poration of GUVs. Before starting the leakage of calcein from the inside of GUVs, the fluorescent intensity remained constant over time, which is considered as the binding state of NPs with the surface of vesicles. The binding of NPs creates surface pressure on the membrane of vesicles. At 3.33 μg/mL NPs, pre-pores converted to transmembrane pore due to the higher surface pressure. Therefore, these descriptions reasonably explained the NPs-induced poration in GUVs as shown in Fig 4. The stochastic pore formation in several GUVs occurred due to the random initiation of pre-pores in the membranes. Such type of stochastic pore formation was observed in antimicrobial peptide-induced pore formation and tension-induced pore formation in GUVs [75, 76]. Due to the repulsive effect at higher sugar concentration, the surface pressure is lower which induces the higher energy barrier for transmembrane pore formation. Hence, the possibility of pore formation decreased with the increase of sugar concentration (Figs 5C and 6C).

As the calcein solution was completely leaked out through the pores, the size of pores would be higher than the size of fluorescent probe, i.e., 0.74 nm. According to the investigation shown in Fig 5, we can consider that the pore formation occurred by the irreversible two-state transition. The first state (intact) is the binding of NPs to the external monolayer of the membrane of GUVs and the second state is the state in which pore is created in the membrane. In the two-state model, the membrane surface bends in a toroidal manner resulting in pore formation [77]. The rate constant, *k*_p_ is defined as the rate of transition from the intact state to the pore state depending on the energy barrier, *U*_B_ given by the Arrhenius equation:

$$k_{p}=A\exp(-\frac{U_{B}}{k_{B}T})$$ (3)

where, *A* is a constant, *k*_B_ is the Boltzmann constant and *T* is the absolute temperature. However, individual events of the two-state transition occur stochastically. If the two-state transition is followed by irreversible pore formation, the fraction of the intact state, *Fr*_intact_ (*t*) can be defined as the fraction of intact GUVs, where no leakage of calcein occurred. After a certain time, there is zero fluorescence intensity found indicating the poration at which calcein leakage occurred. Hence, *Fr*_p_ can be expressed as, 1−*Fr*_intact_(*t*). This type of two-state transition occurred stochastically as shown in Fig 4(D) and 4(F).

The dipole (P^−^−N^+^) of DOPC-GUVs has greater attraction to the anionic NPs than to 40%DOPG/60%DOPC-GUVs [30]. The attractive force between NPs and lipid membranes may reach a critical value causing area mismatch between the outer monolayer and inner monolayer faster, resulting in greater fraction of pore formed DOPC-GUVs than the 40%DOPG/60%DOPC-GUVs. In contrast, the repulsive force between OH^−^ and the anionic NPs in 40%DOPG/60%DOPC-GUVs may cause less attraction than the DOPC-GUVs. Hence, area mismatch may increase with longer time in 40%DOPG/60%DOPC-GUVs. This may cause higher fraction of deformed and lower fraction of pore formed 40%DOPG/60%DOPC-GUVs compared to DOPC-GUVs. This situation is consistent with our investigations and is seen clearly in Fig 7.

## 5. Conclusions

We investigated the anionic magnetite NPs-induced vesicle compactness, vesicle deformation, and lipid membrane poration under various sugar concentrations. The higher concentration of sugar provides additional structural stability to the GUVs, and, consequently, reduces the compactness along with the fraction of deformation and poration. The differences in the NPs effects at lower and higher sugar concentrations are explained by the enthalpy and entropy-driven interactions between sugar molecules and lipid membranes. The area-difference-elasticity model and the classical theory of pore formation, respectively, reasonably explain the deformation and poration of vesicles. The differences in fraction of deformed GUVs and GUVs with pores for DOPG/DOPC and DOPC membranes under various sugar concentrations are explained based on the interaction of NPs with the lipid head structures. The biological implications of sugar molecules as well as their extensive application in biophysical and biochemical research determine the interest in the NPs-induced deformation and poration of cell-sized vesicles under various sugar concentrations. Due to the rapid increase of nano-bio-technology-based research and industry, it is crucial to save ourselves from the exposure of NPs. Adsorption of NPs deforms the cells and also forms pores in cell membranes. Exposure to NPs affects the cardiovascular and pulmonary activities, resulting in substantial mortality and morbidity. These investigations may contribute to the development of new medical and pharmacological technologies with proper safety measurements.
